# Change of perspective: bibliometrics from the point of view of cited references—a literature overview on approaches to the evaluation of cited references in bibliometrics

**DOI:** 10.1007/s11192-016-2111-2

**Published:** 2016-08-20

**Authors:** Werner Marx, Lutz Bornmann

**Affiliations:** 1 Max Planck Institute for Solid State Research, Heisenbergstraße 1, 70569 Stuttgart, Germany; 2 Division for Science and Innovation Studies, Administrative Headquarters of the Max Planck Society, Hofgartenstr. 8, 80539 Munich, Germany

**Keywords:** Reference analysis, Cited references, Interdisciplinarity, Growth of science, Creativity

## Abstract

Citation analyses normally investigate the number of citations of publications (e.g. by people, institutions or journals) where the information on times cited from the bibliographic databases (such as Scopus or Web of Science) is evaluated. But in recent years, a series of works have also been published which have undertaken a change of perspective and are based on the evaluation of the cited references. The cited references are the works cited in the publications which are used to calculate the times cited. Since these evaluations have led to important insights into science and into scientometric indicators, this paper presents an overview of methods based on cited references, and examples of some empirical results from studies are presented. Thus, the investigation of references allows general statements to be made on the precision of citation analyses, and offers alternatives for the normalization of citation numbers in the framework of research evaluation using citation impact. Via the analysis of references, the historical roots of research areas or the works of decisive importance in an area can be determined. References allow quantitative statements on the interdisciplinarity of research units and the overall growth of science. The use of a selection for the analysis of references from the publications of specific research areas enables the possibility of measuring citation impact target-oriented (i.e. limited to these areas). As some empirical studies have shown, the identification of publications with a high creative content seems possible via the analysis of the cited references. The possibilities presented here for cited reference analysis indicate the great potential of the data source. We assume that there are additional possibilities for its application in scientometrics.

## Introduction

Scientific studies essentially consist of two parts: the actual text including illustrations and tables, and the list of cited references. The latter is a listing of the works to which the publication in question refers (on which it is constructed or which it discusses) and which it cites in the form of references (Merton 1965). With the references, earlier works are taken up and integrated into the network of scientific publications via the citing publications. References or citations are the threads which connect the publications formally and in content. Many bibliographic databases use this connection and thus become citation indices: publications and their citations are linked in the citation index and made searchable (as, for example, in the Web of Science, WoS, Thomson Reuters). A particular document appears only once as a document in the database, and then only if it appears in a journal which is covered by the database in question. A publication appears in the database as a reference or a citation as many times as it is cited by other works included there. But the publications included in a database like the WoS are only partially identical with the references cited therein and correspondingly linked. References can refer to works in journals which are not covered in the database, or to books or other publication types not covered. The frequencies used to determine the citation numbers (WoS: times cited) always relate to the subset of linked references (Marx and Bornmann 2015).

**Fig. 1:**
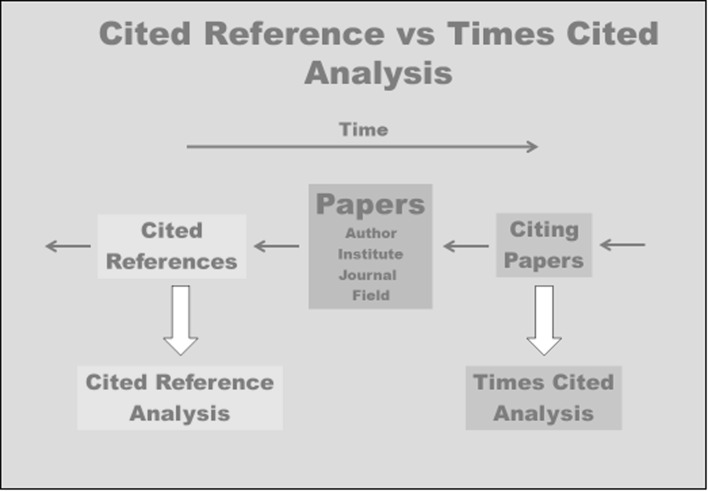
Schematic representation of the connection between the cited reference and the times cited perspective in the bibliometric analysis of a publication set (e.g. the papers of a researcher, a research unit or a journal)

One can now view each scientific work from the citing perspective and determine how many citations this work has received in total (WoS: times cited). But one can also switch to the cited perspective and determine how often this work appears as a reference in other works (Bornmann and Marx 2013). The two perspectives are, of course, closely interconnected. Figure 1 shows schematically the connection between the cited reference and the times cited perspective of a bibliometric analysis. Whereas the cited reference analysis analyses the references cited by the works of a publication set (e.g. the works of a researcher, of a research unit or a journal), the times cited analysis investigates how many publications which appear later have cited the relevant work of the publication set. The cited references have appeared before the work of the publication set, whereas the citing papers have appeared later.

Most of the citation analyses published in recent years investigated the number of citations (Waltman 2015). Thus the times cited information from the databases was evaluated. But a series of works have also been published which have undertaken a change of perspective and are based on the evaluation of the cited references. Since these evaluations have led to important (sociology of science) findings (such as statements on the growth of science) or fulfil an important function in bibliometrics (such as the normalization of citation impact with respect to the subject category), we would like to provide, in what follows, a summary of the most important works which are based on evaluation of cited references. This overview is meant mainly to draw attention to the potential which lies in the evaluation of cited references, and to stimulate additional evaluations on the level of cited reference data.

## Overview of studies which are based on the evaluation of cited references

### Investigation of the precision of citation analyses

The searchable works stored as records in a database are subject to formal criteria so that the records can be found in a literature search via various search fields (such as the author or journal fields). Thus, for example, not only the author or journal names, but also the numerical data of a paper (such as the volume and page numbers) are standardized. The handling of the references is—at least by Thomson Reuters in the WoS—only partially standardized: the references are included in the database exactly as they occur in the citing work; only the names of journals and the titles of works (such as books or reports) are standardized or abbreviated. Mistakes in the numerical data for year, volume or page numbers are not corrected.

The lack of standardization of references has considerable consequences for their handling by the database operators. These may be perceptible if, for example, one performs a search in the WoS on the level of references (cited references search modus). Here one quickly notices that most works are cited in numerous variants which are generally all the more numerous the more often a work has been cited in total. The variants arise partially as a result of the different standards of the journals which work with different styles for the representation of references in a paper. Lack of attention (e.g. typos) also produce citation mutants which are sometimes copied by citing authors and “mutate” further (Buchanan 2006). Older works are subject to the risk of mutation for longer and are therefore generally more affected (Marx 2011). Variants in the references also arise from changes in the general citation behaviour. For instance, it used to be normal not to cite the first page of a work (as is normal today), but a particular page on which a particular statement, illustration or formula can be found. This gives rise to a new variant of a cited work each time.

Thor et al. (2016), for example, have collected all the variants cited by publications which appeared in the *Journal of Informetrics* in Table 1. One of the cited publications is Hirsch (2005), in which the h index was introduced. As the results in the table show, there is a series of variants besides the reference mainly used.

**Table 1:** Variants of the same cited reference (Hirsch 2005). *Source*: Thor et al. (2016).

Cited reference	Number of citations
HIRSCH J, 2005, P NATL ACAD SCI USA, P16569	1
Hirsch J., 2005, P NATL ACAD SCI USA, V102, P165	1
Hirsch J. E, 2005, P NATL ACAD SCI USA, V102, P16569	1
Hirsch J. E., 2005, P NATL ACAD SCI, V102, P16569	1
Hirsch J. E., 2005, P NATL ACAD SCI USA, V102, P16569	1
Hirsch JE, 2005, P NATL ACAD SCI USA, V102, P16569, DOI 10.1073/pnas.0507655102	171
Hirsch JE, 2005, P NATL ACAD SCI USA, V102, P16572, DOI DOI 10.1073/PNAS.0507655102	1
Total	177

Variants of references are formal errors which hinder the technical handling of references and make the collection of citations for a work prone to error. The database document corresponding to a reference is not always recognized as such and linked correspondingly. Citations of database documents are lost more or less often. Thus, the problem of cited references variants leads to the problem of citation linking or matching citations in bibliometrics (Olensky et al. 2015). Cited references with inaccuracies result in missed matches in the WoS and lead to reduced citation counts for papers (times cited information in WoS). Depending on the definition of the errors or deviations in a study, as well as the scope of the data sets investigated, and the age and specialist area of the references, missed matches in the WoS of between 5.6 % (Olensky 2015) and 12 % (Hildebrandt and Larsen 2008) are reported in the literature. Moed (2005) performed “the most comprehensive study on the accuracy of cited references in the WoS to date” (Olensky et al. 2015). He investigated 22 million cited references and the matching to their 18 million target articles and found 7.7 % discrepant cited references resulting in a missed match with WoS target papers. Similar results were found in other studies (Franceschini et al. 2016).

Non-matched citations in the WoS are a significant source of error for any citation analysis, e.g. for research assessment. Thus, solutions are wanted—as offered for example in the application CRExplorer (www.crexplorer.net)—which contribute to reducing the number of variants of the same cited reference in bibliometric data. But since it will probably never be possible to avoid all missed matches in a database, the studies into the scope of the missed matches provide important indications on the accuracy and the interpretation of bibliometric data. The studies make it clear that there is little sense in providing bibliometric data with great precision, i.e. with several digits after the comma (as is done in the Journal Impact Factor of Thomson Reuters, for instance) or to interpret at this level of accuracy (Hicks et al. 2015). Instead, one should either work with confidence intervals or stability intervals (Bornmann et al. 2013; Waltman et al. 2012) to obtain indications of the uncertainty of bibliometric data, or one should assume correspondingly great deviations in the interpretation of the results.

### Normalization of citation numbers

**Fig. 2:**
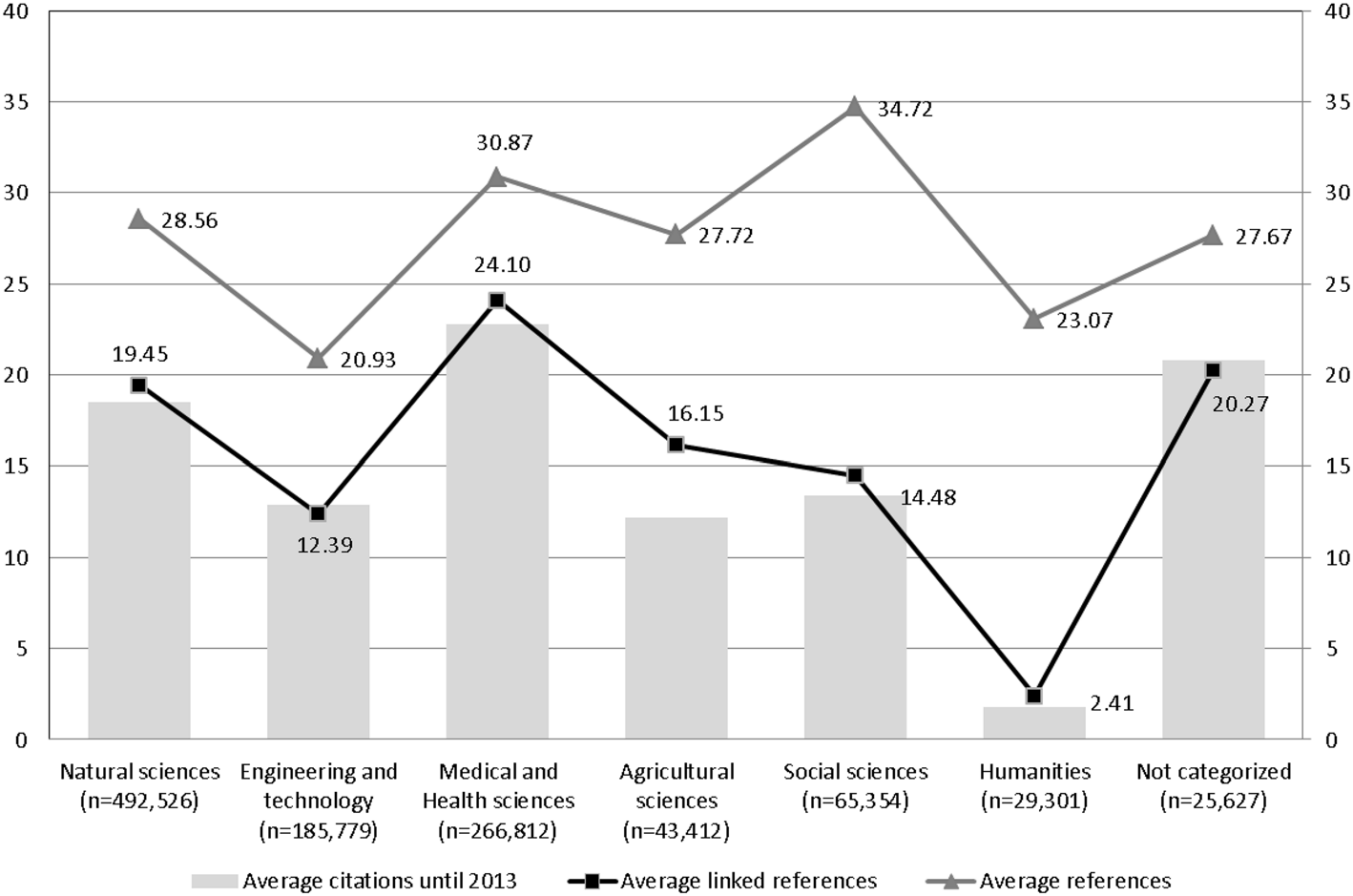
Average number of citations from publication year until the end of 2013 (*grey bars*), average number of cited references (*triangles*), and average number of linked cited references (*squares*) of articles published in 2005. The articles have been categorized into disciplines by using the OECD category scheme which corresponds to the Revised Field of Science and Technology (FOS) classification of the Frascati Manual. *Source*: Marx and Bornmann (2015)

Evaluative bibliometric studies are mostly based on the evaluation of the times cited information in the bibliographic databases (see above). Here the total number of citations for a particular work (or the works of an author, a research institution, a journal etc.) is determined. Since the works investigated often come from different publication or citation cultures, their citation numbers are not comparable with one another. For example, the citation of a work in mathematics has a higher weighting than the citation of a work in biochemistry. The reason for this is the different average number of references in the two subjects: in biochemistry there is a higher average number than in mathematics. As Marx and Bornmann (2015) have determined, the subject areas differ mainly in regard to the cited references which are linked with a database document. As shown in Fig. 2 of the publication by Marx and Bornmann (2015), the papers from the area of humanities have an average of 23.07 cited references. Limiting the cited references to those which are also linked in the WoS, reduces the average to only 2.41. In some subjects (such as medical and health sciences) the coverage (and thus the linking) is greater than in other areas (such as social sciences).

The lack of comparability of citation numbers between the subject areas in a bibliographic database (such as the WoS) has led in bibliometric practice to the standardization of citation impact with normalizing procedures—an overview of different methods can be found in Bornmann and Marx (2015) and Waltman (2015). Only after normalization can evaluations be performed of research units which have published papers in different subject categories. The current standard in bibliometrics is represented by the normalization procedures which are used in evaluative bibliometrics as cited side normalization to establish the comparability of citation numbers. They are based on the following principle: the citation number of a particular work is compared with the citations found for a comparable publication set (all works from the same subject category and the same publication year). The citations found form the expected value. In the comparison it is determined how strongly the work in question deviates upwards or downwards from the expected value (or the reference value).

In recent years a new method has established itself, known as citing side normalization, alongside the cited side normalization procedure. This method considers the citation environment of a citation by weighting the citation depending on the field it comes from. Thus, the citations are not compared in total with an expected value, but each individual citation receives a field-specific weighting. This weighting is performed via references: each citation is divided by the number of references which the citation culture in a subject area should reflect. In the simplest approach of citing side normalization, each citation is divided by the particular number of references in the citing work. Since the individual citing works may not be typical for the whole of the field, the weighting factor can alternatively be based on the average number of references of all works in the particular journal in which the citing work has appeared. One can assume that this better reflects the citation culture of a field than with the cited references of only one paper.

This approach of citing side normalization can be refined further by considering only the linked references (that is, only those references which refer to existing database documents). Because their share is more typical for the times cited information in the database than the average number of references overall (which varies much less, see Marx and Bornmann 2015). In the calculation one can additionally consider only a particular citation window by limiting the basis to the average number of references for a particular time window. One hereby allows the indicators to be influenced by the time dependence of the average number of references in the fields: in some fields the more recent literature is referred to more often and in other areas the older.

According to the empirical results of Waltman and van Eck (2013), citing side normalization is superior to cited side normalization: it is said to counteract the field dependency of citations better. In addition, citing side normalization offers a decisive advantage compared with cited side normalization: there is no need to collect a reference set to represent the field of the cited publications. The problem with the definition of references sets is that, although it is a more or less common practice, there are no standards. Doing without reference sets thus leads to a simplification of the calculation of normalized impact scores.

### Target-oriented measurement of citation impact

The citations which appear in bibliographic databases for a work (times cited) generally do not arise just from citing works in the same field as the cited work, but from very varied fields. Although the studies which present the results of citation analyses often assert that they measure the impact in a particular field, this is generally not the case. For example, “the result is the identification of high performers within a given scientific field” (Froghi et al. 2012, p. 321). “Ideally, a measure would reflect an individual’s relative contribution within his or her field” (Kreiman and Maunsell 2011). “That is, an account of the number of citations received by a scholar in articles published by his or her field colleagues” (Di Vaio et al. 2012, p. 92). The cited side normalization procedure described in “Normalization of citation numbers” section does take account of the field of the work to be evaluated, but not that of the citing work. This approach results in a certain imprecision with regard to the quantification of citation impact and thus the significance of cited works: ultimately it is unclear which impact is actually involved. In the extreme case, a publication which is often cited could have achieved its impact solely in other fields and none in its own.

Bornmann and Marx (2013) have therefore suggested expanding the perspective of evaluative bibliometrics and to augment the normally used times cited based analysis with a cited reference based analysis, and to measure the research-area specific impact with the cited reference based analysis. For the precise delineation of the field, they have used a (single) chemical compound: as an example, all the references of the publications were extracted and investigated, which deal with the medicine Aspirin and were included in the field-specific bibliographic database of the American Chemical Abstracts Service (CAS). The cited references were examined to find which works and authors were particularly often cited by colleagues. On the basis of the cited journals, their significance within the publication set (i.e. the research area delimited by Aspirin) is determined and quantified in the form of field-specific journal impact factors (number of cited references divided by number of publications). The result is shown in Table 2: the 10 journals with the highest number of cited references in the area of Aspirin research are listed there.

**Table 2:** Journal citation impact on Aspirin research. *Source*: Bornmann and Marx (2013)

Journal	Number of cited references in 2010 (with reference publication years 2007 and 2008 only)	Number of publications in 2007 and 2008	Journal Impact Factor: number of cited references divided by number of publications
*Journal of the American College of Cardiology*	357	29	12.31
*Circulation*	261	8	32.63
*New England Journal of Medicine*	209	5	41.80
*Thrombosis and Haemostasis*	153	46	3.33
*European Heart Journal*	150	34	4.41
*Journal of Thrombosis and Haemostasis*	139	31	4.48
*Lancet*	120	20	6.00
*American Journal of Cardiology*	94	49	1.92
JAMA, the *Journal of the American Medical Association*	87	5	17.40
CHEST	84	19	4.42

The journals are sorted by the number of cited references. The ten journals with the highest number of cited references are shown

A change to the evaluation of the cited references (instead of the times cited) generally offers decisive advantages in the target-oriented measurement of citation impact. With the cited references analysis, the impact within a clearly defined publication set can be determined where the recipient of impact is specified unambiguously. Here the recipient can look very different: the work of an author, a journal, a field or also a particular research topic (see above). The analysis can also be used to check, for example, how strong the citation impact of selected works is on a particular research area in Europe or a research organization in the USA. Finally, one can investigate aggregates such as the cited authors or journals instead of (or as well as) individual references, and thus measure their weighting within a selected publication set (such as a particular research field).

The change suggested by Bornmann and Marx (2013) from times cited to cited reference analysis may perhaps appear trivial; but it is this change which first allows an exact determination of which recipient of impact bibliometric analysis one is actually dealing with.

### Historical roots

Marx et al. (2014) have recently suggested the Reference Publication Year Spectroscopy (RPYS) method, with which the historical roots of a research area can be investigated. Since this method is relatively simple to use and leads to interesting historical insights into a research area, it has already been applied many times: Leydesdorff et al. (2014) have applied the RPYS to the historiography of i-Metrics (i.e. bibliometrics, scientometrics, informetrics, and webometrics), that is, to the field in which the method was developed. They have identified the pioneering researchers (such as Lotka, De Solla Price, Kessler, Garfield) and the decisive works to which the community mainly refers. Wray and Bornmann (2015) have investigated the historical roots of the philosophy of science and determined among other things that Einstein’s work on the special theory of relativity plays a special role in this area.

Marx and Bornmann (2014) were able to show, with the example of the Darwin finches, that the RPYS is in a position to determine the origin of scientific legends and myths. Comins and Hussey (2015a) have identified those works which were of decisive importance for the development and technology of the Global Positioning System (GPS). Elango et al. (2016) have done the same for the area of tribology (lubricants). Finally, Gorry and Ragouet (2016) have investigated the historical roots of a medical field at the interface between cardiology and radiology (interventional radiology). Two applications were recently presented, with which RPYS could be performed with software support (see www.crexplorer.net and www.comins.leydesdorff.net).

The RPYS is based on the analysis of the publication year of the references which were cited by the publications of a research field. The method is based on finding important earlier works which contain the historical origins or the intellectual roots of the research field in question (that is, the shoulders on which today’s giants stand). Use is made here of the fact that such works are relatively often cited by the relevant community. There is a kind of community coordination on which works from the past to invoke (Bornmann and Marx 2014). If one pictures the frequency of the cited publications (the cited references) as a function of their publication year, such works appear as striking peaks. Especially in the early years, influential works can easily be identified, since the peaks contain many references to individual (highly cited) publications.

**Fig. 3:**
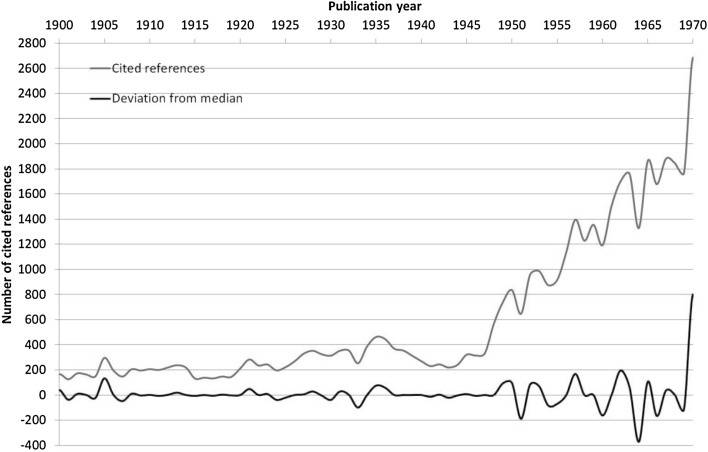
Reference Publication Years Spectroscopy (RPYS) of four journals relevant for the philosophy of science: *British Journal for the Philosophy of Science*, *Erkenntnis, Philosophy of Science*, and *Studies in History and Philosophy of Science.* *Source*: Wray and Bornmann (2015)

Figure 3, for example, shows the result of the RPYS which was published by Wray and Bornmann (2015) on the historical roots of the philosophy of science. Whereas the blue line shows the annual number of cited references, the red line shows the deviation of the number of cited references in one year from the median of bordering years. For the year 1905 a clear peak is visible, which relates to Einstein’s work on the special theory of relativity and thus indicates the special role of this work for this area.

The analysis of the reference publication years by means of RPYS can be performed not only for the publications of a field but also for the publications of authors and journals: which are the cognitive roots of scientists or to which early works do the authors in a journal frequently refer? Do scientists or journals have a cognitive basis of a few publications which are mentioned very frequently in the literature? There is also the possibility of investigating comparatively which significance individual publications have for particular authors, in particular fields or for particular journals: What impact does the same work achieve in different contexts (Comins and Hussey 2015b)?

### Growth of science

An important advantage of reference analysis is that the cited publications can go back significantly further than the citing publications. Access to the citing publications is limited by the temporal coverage of the bibliographic databases. The databases (such as the WoS) generally reach back no further than the start of the twentieth century. Many field-specific databases (such as GEOREF, Geological Reference File) cover only the period since 1960 or even later. But the references which are cited in publications listed in the databases are not limited in time. The lack of limitation therefore opens the possibility of making historical statements on the whole of science or on subsets indirectly via the references (Marx and Cardona 2004).

The growth of science can be partially quantified by the annual increase in the number of scientific publications. A series of investigations in very different fields has already been published on this. As just one example, reference is made to the pioneering investigations of De Solla Price (1965, 1986). Since the growth of science is also reflected in the increasing number of the cited publications, these investigations also evaluated cited references (e.g. De Solla Price 1965; Tabah 1999; Van Raan 2000; Larivière et al. 2007; Bornmann and Mutz 2015). The evaluations on the growth of science are then based on the part of the publications which were cited at least once. De Solla Price (1965) has already discussed in his book *Little Science*—*Big Science* the growth of science on the basis of references, and later presented empirical results on this (De Solla Price 1986). He could show that science is growing exponentially and that the volume of publications is doubling every 10–15 years. The exponential growth of science was confirmed by subsequent analyses (see e.g. Tabah 1999) and is now a generally accepted fact.

**Fig. 4:**
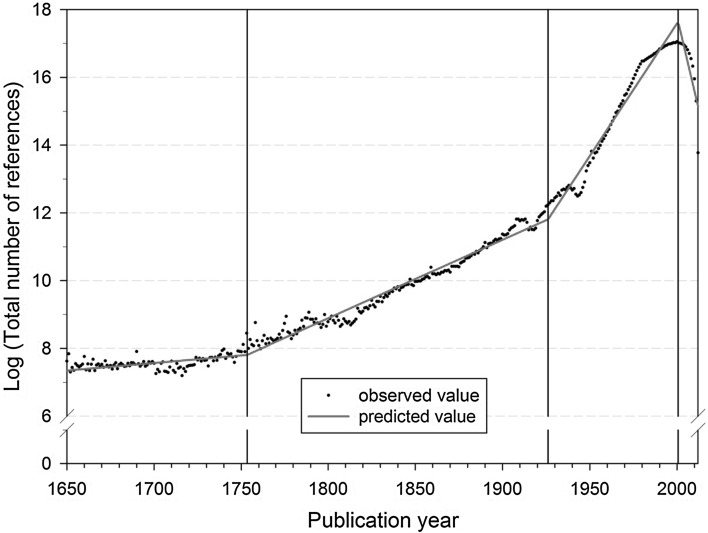
Segmented growth of the annual number of cited references from 1650–2012 (citing publications from 1980–2012). *Source*: Bornmann and Mutz (2015)

Van Raan (2000) has developed a model for the ageing of scientific literature based on the time distribution of all references extracted from the publications of the year 1998, and investigated the growth of science since the start of the twentieth century with it. For example, his empirical results show that science shows a final increase of growth between about 1984 and the end of the 1990s. Larivière et al. (2007) have investigated the half-life period of citations over more than 100 years, and found that, against widespread expectations, the average age of references has risen continuously since the mid-1960s. To put it another way: the closer to the present, the more references there are to older literature. They interpret this as the result of scientific literature no longer growing exponentially. Bornmann and Mutz (2015) have investigated the publications included in the WoS from the period 1980–2012 as well as the publications cited therein (1650–2012) by means of segmented regression analysis. The authors have undertaken a comparison of the results on the growth of science which are based on the number of publications or the number of references, respectively. The reference-based analysis produced a significantly higher growth for modern science than that based on the number of publications appearing annually. Whereas the annual number of publications covered by the WoS since 1980 doubled only every 24 years, the doubling period on the basis of references was only about 10 years (see Fig. 4).

### Interdisciplinarity of research units

Interdisciplinarity has become a significant characteristic of modern science: (1) Many ground-breaking discoveries have been made at the interface between different disciplines (see e.g. Marx and Bornmann 2013). (2) Scientific cooperation activities (measured by co-authorships) are still increasing (Waltman et al. 2011) and increasingly cross field boundaries (Porter and Rafols 2009). In order to measure interdisciplinarity in bibliometrics, it is first necessary to classify papers by field. Three main procedures for this have established themselves in bibliometrics. In order to assign the publications to individual fields, either (1) the publishing journals are grouped by field (Leydesdorff and Bornmann 2016), (2) publications are classified by experts in field-specific databases (such as Chemical Abstracts) (Bornmann et al. 2013), or (3) direct citation links are used to classify the publications with the help of citation data. All procedures attempt to allocate each publication unambiguously to a subject category (Waltman and van Eck 2013). From the diversity of categories for the publications of different research units (such as research facilities or scientists) one can determine how interdisciplinary a unit is.

However, all three procedures indicate weaknesses in the field classification: (Ad 1) If publications are classified by journal sets, there is the area of multidisciplinary journals (such as *Nature* and *Science*). For the publications appearing in these journals, there is no unambiguous field classification. (Ad 2) The classification of publications in field-specific databases always refers to a very limited set of publications. This kind of classification is therefore hardly usable for a study of interdisciplinarity, since the overlapping field categories are missing. (Ad 3) In the use of direct citation links to classify a publication by field, the problem arises that the clusters formed are very difficult to provide with a field label. This means that the method may be able to form field clusters, but the clusters cannot (always) be assigned unambiguously to a particular field label.

Because of these difficulties shown by the common procedures for field classification in bibliometrics, classification via the cited references offers an interesting alternative. In recent years, a series of papers has already used references to measure the interdisciplinarity of research units (such as research fields, journals or authors) (e.g. Larivière and Gingras 2010; Zhang et al. 2009, 2010, 2015). Here, interdisciplinarity is operationalized over the diversity of the subject categories to which the cited references are assigned. For example, one possibility consists in assigning references to the subject categories to which the journals publishing the cited works belong (multidisciplinary journals excluded).

Zhang et al. (2015) have used the Leuven-Budapest subject-classification scheme (ECOOM) for this, which is constructed hierarchically. The more often a research unit has cited different subject categories, the more the research unit’s orientation is interdisciplinary. If a unit is based on work from different fields, their research should be positioned as interdisciplinary. For the measurement of diversity, various mathematical approaches are discussed in the literature (e.g. following Rao-Stirling or Hill-type). Unlike earlier publications by Zhang et al. (2009, 2010) and others, the study by Zhang et al. (2015) measured interdisciplinarity on the basis of individual cited works and not on the basis of the cited journals.

**Fig. 5:**
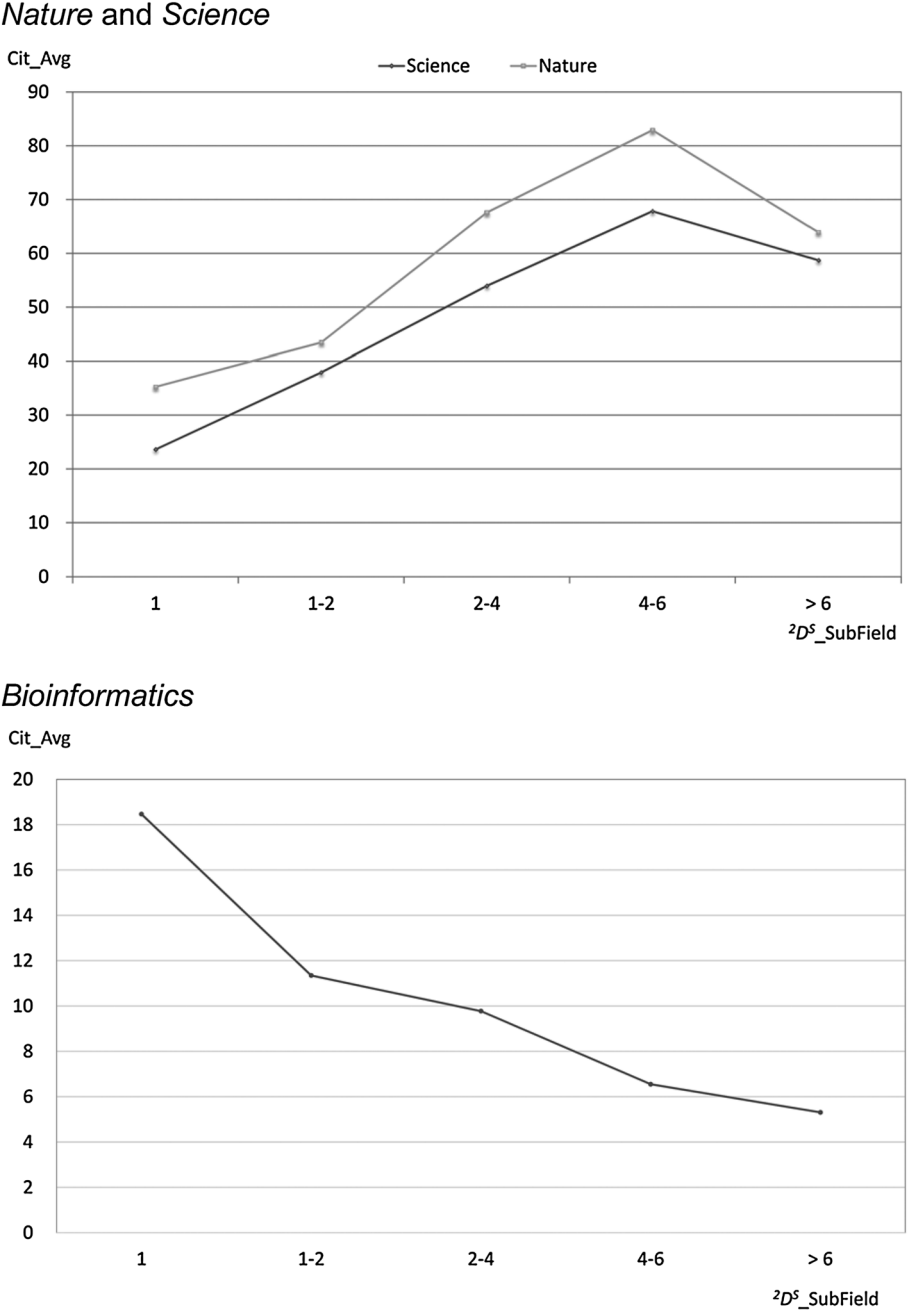
Citation impact (average number of citations) as a function of interdisciplinarity, measured with Hill-type true diversity (^2^D^S^_SubField) for three journals. *Source*: Zhang et al. (2015)

The objective of the studies on interdisciplinarity is not only the discovery of suitable approaches for the measurement of interdisciplinarity on the basis of different units of cited references (such as journals or individual publications), but also the investigation of the connection between interdisciplinarity and citation impact. Thus, for example, the study of Zhang et al. (2015) found quite different correlations between interdisciplinarity and citation impact depending on the journal investigated (see Fig. 5). The multidisciplinary journals *Nature* and *Science* show a continuous increase in the average number of citations with increasing interdisciplinarity, falling off again above a limiting value. The journal *Bioinformatics*, as an interdisciplinary journal, shows quite a different correlation: the citation impact of their publications falls with increasing interdisciplinarity.

### Identification of publications with creative approaches

Creativity arises from the interaction of three elements: (1) a culture which comprises symbolic rules, (2) a person or publication who/which brings something new into the symbolic domain, and (3) a community of experts who recognize and confirm this innovation. Creativity is the cultural counterpart of genetic mutation which underlies biological evolution. Evolution is based on a passive change process: random genetic mutations turn out favourable or unfavourable. But creativity is an active process of change which is directed towards a particular objective. New creative approaches are often only adopted by the colleagues in a field slowly and with a time delay, because science tends to react conservatively and fields are becoming increasingly narrow. New creative approaches are first treated critically and can (generally) only establish themselves after a longer controversy in the field. The increasing specialization in the disciplines leads today to new ideas not always being (immediately) recognized or often not being able to be understood initially (Heinze 2013).

Bibliometrics—or cited reference analysis—has been recognized in recent years as a method which could allow one to measure new creative approaches in publications. If there were a method with which these approaches could be identified early, one could pay specific attention to these approaches in a field. In order for bibliometrics to identify especially important works in a field, citations are generally used. In the WoS, papers which receive relatively many citations shortly after publication are known as “hot papers”; papers which, in the course of time, receive considerably more citations than comparable papers, are known as “highly cited papers”. But the “hot papers” or the “highly cited papers” cannot always be assumed to be papers with creative approaches: (1) The study of Van Noorden et al. (2014), in which the 100 most cited papers since 1900 in the WoS were evaluated, shows “that many of the world’s most famous papers do not make the cut. A few that do, such as the first observation of carbon nanotubes … are indeed classic discoveries. But the vast majority describe experimental methods or software that have become essential in their fields” (p. 550). (2) Since citation-based evaluations can only be undertaken quite some time after the publication of a work, it is not possible to identify important papers with citations shortly after publication.

Uzzi et al. (2013) have presented a method for identifying works with creative approaches not long after the publication of the papers. The authors assume that those publications best spread new ideas which offer a balanced mixture of established knowledge and new ideas. They point to the main works of Newton and Darwin, in which the ground-breaking ideas were presented in connection with generally accepted mathematical methods (Newton) and knowledge of breeding (Darwin). Uzzi et al. (2013) undertook to measure the novelty of current publications using jointly cited (co-cited) references. For this they determined the extent of new field combinations of references for publications.

The field combinations were determined from journal pairs (in other words, from the pairs of journals in which the cited references appeared). In order to determine the new field combinations, they compared the frequency of co-cited references with statistically determined expected values: “We counted the frequency of each co-citation pair across all papers published that year in the WOS and compared these observed frequencies to those expected by chance, using randomized citation networks. In the randomized citation networks, all citation links between all papers in the WOS were switched by means of a Monte Carlo algorithm“(Uzzi et al. 2013, pp. 468). Those works which are based on especially many unusual combinations should have an especially high novelty value and thus be especially interesting for readers in a field.

**Fig. 6:**
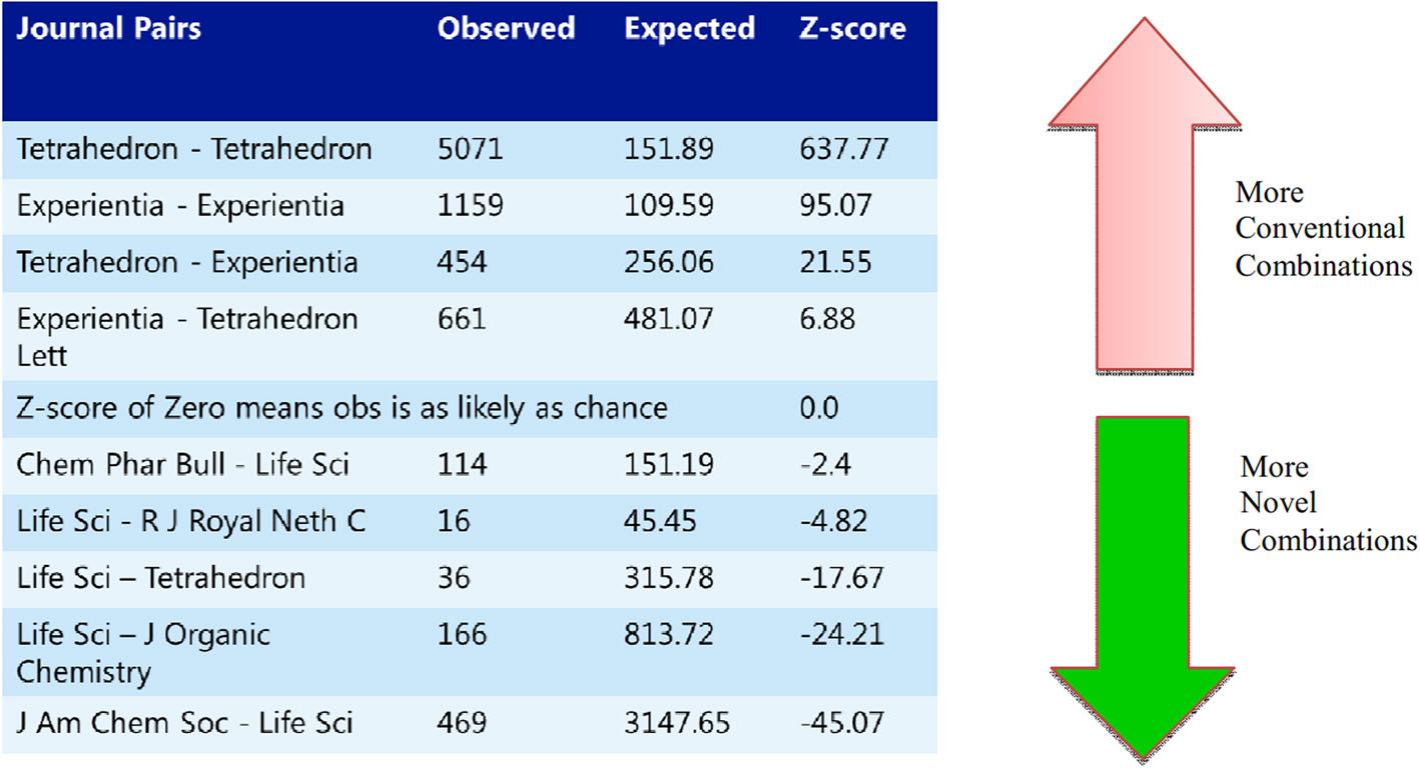
Examples of journal pair frequencies for the paper “Synthesis of the Five Natural Cannabis Spirans”. *Source*: Uzzi et al. (2013), supporting information

Figure 6 shows examples of more conventional or more novel combinations of journal pairs, which appeared in the paper “Synthesis of the Five Natural Cannabis Spirans”. In order to compare the observed value of the journal combination with the expected value, a z-score was calculated for each pair. The higher the z-score, the more conventional is the combination; low z-scores indicate more novel combinations. On the basis of 18 million publications from all disciplines in the WoS, Uzzi et al. (2013) determined that works with unusual combinations have twice the probability of later becoming highly cited papers than works with conventional combinations. This method thus seems to offer a very promising possibility of identifying new, creative approaches in a field relatively soon after a paper is published.

Whereas Uzzi et al. (2013) have looked at the atypicality of referenced journal combinations, in order to identify creative approaches in papers, Wang et al. (2016) followed a different methodical approach: they focus „specifically on novelty of referenced journal combinations, examining whether an article makes new referenced journal combinations which have never been made in prior publications“. A similar approach to Wang et al. (2016) is being followed by Warman et al. (2013). However, they did not evaluate the novelty of referenced journal combinations, but the novelty in MeSH term combinations. In agreement with the results of Uzzi et al. (2013), Wang et al. (2016) found that „highly novel papers, defined to be those that make more (distinct) new combinations, have more than a triple probability of being a top 1 % highly cited paper when using a sufficiently long citation time window to assess impact”. According to Glänzel and Schubert (2016), the three approaches for the identification of creative approaches in publications can be attributed to what Koestler (1964) called bisocitation: “The perceiving of a situation or idea in two self-consistent but habitually incompatible frames of reference”. Creativity thus arises when two well-known, but yet unconnected entities are linked to one another.

## Conclusions

The change from the times cited to the cited reference perspective offers a whole range of benefits for bibliometric analyses, which have already been used by many studies. In this paper we have made the attempt to present areas where cited reference analysis has profitably been used. Investigations into the error rate of references allow statements on the precision of citation numbers which are used preferentially in research evaluation and should be scrutinized especially thoroughly as sensitive data. The use of citing side normalization of citation numbers offers the possibility of doing without reference sets in the normalization. The RPYS method allows the determination of the historical origins or the intellectual roots of research fields by use of bibliometric data. This may not replace the work of historians, but facilitate their investigations and make statements on the significance of earlier works quantifiable. Also the quantification of the growth of science by the evaluation of the cited references can be used very well in studies on the history of science. The measurement of the target-oriented citation impact, of the interdisciplinarity of research units as well as the identification of creative approaches in publications are further examples for the great potential of cited references analysis.

Cited reference analysis opens additional possibilities going beyond the approaches and methods presented so far, which—as far as we know—have hardly been applied or explored. Thus cited references can be used to measure or quantify the field-specific historicity of fields. Here one determines how high the share of older references (e.g. from works which appeared before the twentieth century) in the field-specific publications is, or how the mean referenced publication year differs by field. Our own analyses first showed that the works cited by the community in question in their publications reach back to different extents into the past: modern fields with a shorter history, such as molecular genetics, cite relatively few older works (e.g. from the nineteenth century), whereas mathematics or the social sciences and humanities cite relatively many early works. The strength of the “memory” for the scientific past and the use of findings from different time periods thus seem to vary with the field.

The reconstruction of the evolution of a research field could be another area in which the evaluation of cited references leads to interesting results. Here it is determined for each cited reference in the publication set of a field how many citations it has received over the years. The number of citations per year is compared with the average number of citations which the references from the same cited reference year have received. Above average citation numbers indicate references (or publications) which have exercised a significant influence on the research field over the whole or a specified part of the time period. Thus it can be determined which works have played a special role in the individual publication years up to the present. With this method, the evolution of a research field can be reconstructed from the references cited in the relevant publications. Thus the publishing community in a research field “votes” (Bornmann and Marx 2014) on which works have earned the greatest attention in the particular years and were most important for the particular research field.

We could imagine that in particular the impact measurement of publications within clearly delimitable research fields will be of special interest in future. Research evaluation is the main application area of bibliometrics. Target-oriented impact measurement would allow the effect of individual works, authors or institutions to be measured within specified publication sets. The effect of a work beyond its field is, of course, interesting and even desirable. It is generally the ground-breaking works which are cited well beyond their area of origin. But there is often the desire in research evaluation to be able to make an exact statement about the citation impact of a publication set. Thus, for example, one could determine which publications, authors or institutions exercise an especially great impact on the research in a country or of an institution.

References which are cited together (co-cited) are mostly related in content. Analyses of the relatedness of publications on the basis of co-citations have become a much used bibliometric procedure. The RPYS opens the possibility of investigating the citation environment of a specific work (or combinations of particular works) by co-citations: One learns which works from which years are cited how often together with the selected (and co-cited) work. The specific reference should be a prominent and seminal early work which can be used as a kind of marker or tracer reference for the research topic under study. We may assume that papers which cite the selected reference(s) are potential candidates for citing also most other references relevant in the specific historical context (Marx et al., in preparation). In addition, one can discover, for example, whether any—and if so which—works exist leading up to a particular pioneering work. The contributions of precursors which stimulated the development of new methods or theories can easily be forgotten. But the evaluation of the references in the publications of the corresponding specialist community can reveal such works. A certain subset of the colleagues who cite an especially prominent pioneer work have indeed also cited the precursor works which are often later forgotten.

In this work we have presented a series of possibilities for how bibliometrics could look when based on cited reference analysis. With this work we would like to indicate the potential of these analyses and encourage their application. Many of the methods which are used for cited reference analysis are not yet mature and can purposefully be developed further in future studies.
